# SIRT7 upregulation protects against TKI-induced cardiotoxicity by inhibiting MCU-mediated mitochondrial calcium overload

**DOI:** 10.3389/fphar.2026.1929231

**Published:** 2026-09-18

**Authors:** Meiling Gao, Zhe Wang, Zhiling Cheng, Suhua Qiu, Wenting Wu, Zhihan Zhang, Wei Yan, Xianying Wang, Yanfang Xu, Congxin Li

**Affiliations:** 1 Department of Pharmacy, Hebei Medical University Third Hospital, Shijiazhuang, Hebei, China; 2 Hebei Medical University, Shijiazhuang, Hebei, China; 3 Department of Nutrition, The Fourth Hospital of Hebei Medical University, Shijiazhuang, Hebei, China; 4 Hebei Provincial Key Laboratory of Precision Anesthesiology and Organ Protection, Shijiazhuang, Hebei, China

**Keywords:** calcium homeostasis, cardiotoxicity, human induced pluripotent stem cell-derived cardiomyocyte, mitochondrial calcium uniporter, sirtuin 7, tyrosine kinase inhibitor

## Abstract

**Background:**

Tyrosine kinase inhibitors (TKIs) are potent anticancer agents; however, their clinical application is often restricted because of cardiotoxic effects. The mechanisms underlying cardiotoxicity that are shared across different TKIs remain inadequately understood.

**Purpose:**

This study aimed to investigate the role of sirtuin 7 (SIRT7) in regulating mitochondrial calcium homeostasis during TKI-induced cardiomyocyte injury and determine whether the SIRT7–mitochondrial calcium uniporter (MCU) axis could serve as a common protective target.

**Methods:**

AC16 cells were treated for 72 h with three TKIs: sunitinib (4.11 µM, IC_30_), trametinib (12.21 µM, IC_30_), and imatinib (21.84 µM, IC_30_). Cell viability, cardiomyocyte injury markers (N-terminal pro-brain natriuretic peptide and cardiac troponin I), cytosolic and mitochondrial Ca^2+^ levels, mitochondrial membrane potential, adenosine triphosphate (ATP) content, and endoplasmic reticulum–mitochondria contacts assessed using transmission electron microscopy were evaluated. Proteomic analysis was performed to identify differentially expressed proteins shared across treatments. In human induced pluripotent stem cell–derived cardiomyocytes (hiPSC-CMs), contraction amplitude was measured using an impedance-based system, and mitochondrial Ca^2+^ overload was evaluated.

**Results:**

All three TKIs induced concentration-dependent injury in AC16 cells, accompanied by mitochondrial Ca^2+^ overload, ATP depletion exceeding 50%, and increased mitochondria–endoplasmic reticulum (ER) contacts. Proteomic analysis identified consistent SIRT7 downregulation across the three TKI treatments. SIRT7 overexpression mitigated TKI-induced cellular damage, reversed MCU upregulation and functional activation, restored mitochondrial function, and reduced excessive mitochondria–ER contacts. In hiPSC-CMs, SIRT7 overexpression reduced mitochondrial Ca^2+^ overload and prevented TKI-induced contraction inhibition.

**Conclusion:**

SIRT7 upregulation protects against TKI-induced cardiotoxicity by inhibiting MCU-mediated mitochondrial calcium overload.

## 1 Introduction

Tyrosine kinase inhibitors (TKIs) are important targeted anticancer drugs that block abnormal signal transduction by inhibiting kinase activity (Force and Kolaja, 2011). However, their cardiotoxic effects such as heart failure and arrhythmias are becoming increasingly prevalent, limiting their clinical use (Gharwan and Groninger, 2016; Shyam et al., 2023). Recent findings suggest that TKIs lack tyrosine kinase–targeting specificity and may disrupt cardiac homeostasis by inhibiting kinases shared by the heart and tumors (on-target effects) or by inhibiting kinases that are not antitumor targets (off-target effects), resulting in cardiac toxicity. The cardiotoxicity mechanisms among TKIs share common features, such as induction of mitochondrial dysfunction, endoplasmic reticulum stress, calcium homeostasis imbalance, and hERG channel inhibition (Wu et al., 2024). However, they also exhibit diversity owing to their distinct targets. For example, sunitinib (Snt) causes myocardial ischemia, third-generation epidermal growth factor receptor (EGFR)-TKIs cause QT prolongation, and imatinib (Ima) interferes with myocardial repair. The point of convergence of these mechanisms lies in the disruption of the endoplasmic reticulum–mitochondrial axis (Bertero and Maack, 2018; Li et al., 2019). Moreover, cardiomyocyte contraction is highly dependent on adenosine triphosphate (ATP) supply and on excitation–contraction coupling (ECC), which is driven by dynamic changes in intracellular Ca^2+^ concentrations (Landstrom et al., 2017). Notably, TKIs disrupt Ca^2+^ homeostasis in cardiomyocytes, triggering endoplasmic reticulum stress, reactive oxygen species release, and mitochondrial dysfunction, leading to myocardial injury and dysfunction.

Sirtuin proteins are a family of nicotinamide adenine dinucleotide (NAD^+^)-dependent enzymes that perform deacetylation. They also play a key role in metabolism and stress adaptation. In mammals, seven sirtuin isoforms have been identified, each of which exhibits distinct subcellular localization and functions (Crow, 2004; Chen J. W. et al., 2023). SIRTs are involved in various heart diseases, including cardiac hypertrophy, fibrosis, ischemic injury, cardiotoxicity, and heart failure (Cozzolino et al., 2025; Yang et al., 2019). Specifically, nuclear-localized sirtuin 7 (SIRT7) is involved in the regulation of mitochondrial function and cardiomyoprotection, and its absence can exacerbate cardiac injury and lead to cardiomyopathy (Ryu et al., 2014; Vakhrusheva et al., 2008; Winnik et al., 2015).

Although extensive research has been conducted on the mechanisms of cardiac toxicity associated with individual TKIs, the underlying shared mechanisms remain unclear (Shim et al., 2017). Nilotinib, vandetanib, and mobutinib have been reported to induce arrhythmias by inhibiting WNK1 kinase (Wu et al., 2024). Snt causes calcium regulation disorders and contractile dysfunction by inhibiting the PI3K–SGK1 pathway (Li et al., 2019; Gao et al., 2025), suggesting that different TKIs may cause cardiotoxicity through shared key targets or pathways (Dobbin et al., 2021; Leiva et al., 2023).

This study builds on previous findings by selecting three TKIs with distinct targets and mechanisms of action (Snt, Ima, and trametinib [Tra]) to investigate the common mechanisms by which TKIs induce cardiac toxicity via calcium dysregulation. We examined their common features in inducing myocardial injury using the AC16 cardiomyocyte model and used proteomics to identify shared critical regulatory molecules and validate their potential protective effects. The protective effects of SIRT7 upregulation against TKI-mediated contraction inhibition were investigated in human induced pluripotent stem cell–derived cardiomyocytes (hiPSC-CMs). This study aimed to provide a theoretical basis for the clinical prevention and treatment of TKI-induced cardiotoxicity, provide a foundation for the development of targeted protective strategies, advance precision medicine in oncocardiology, and achieve an appropriate equilibrium between anticancer efficacy and cardiovascular safety.

## 2 Methods

### 2.1 Cell culture

AC16 cells (Servicebio, Wuhan, China, STCC13101P-1) were cultured in Ham’s F-12 nutrient mixture/Dulbecco’s modified Eagle’s medium (F12/DMEM) basic (Gibco, Carlsbad, CA, United States; 11320033), with 10% fetal bovine serum (Gibco, United States; A5256701) and 100 μg/mL penicillin–streptomycin (Gibco; 15140163). hiPSC-CM cells (Ronovation, Hangzhou, China; RONGYINNO102012) were cultured in iPSC-CM culture medium (Ronovation; P0901007). Cells were cultured in a constant temperature incubator at 37 °C under an atmosphere containing 5% CO_2_. The cells were then divided into the following groups according to the experiment: plasmid transfection with SIRT7 (Miaolingbio, Wuhan, China; P45894), RU360 (Sigma-Aldrich, Saint Louis, MO, United States; 557440), and with or without Snt (MCE, Monmouth Junction, NJ, United States; HY-10255A), Tra (MCE; HY-10999), and Ima (MCE; HY-15463).

### 2.2 Clinically relevant exposure concentrations

To contextualize the *in vitro* exposure concentrations used in this study relative to clinically achievable levels, we reviewed the pharmacokinetic profiles of the three TKIs. Snt is administered orally at 50 mg daily on a 4-week-on/2-week-off schedule (or as continuous daily dosing). At steady state, the maximum plasma concentration (C_max_) of Snt ranges from approximately 63.6–71.5 ng/mL, whereas that of its primary active metabolite, SU12662, ranges from approximately 19.4–27.5 ng/mL; thus, the total active drug (sunitinib plus SU12662) C_max_ reaches approximately 82.4–98.3 ng/mL (approximately 0.3–0.35 µM). Snt and its active metabolite are approximately 95% and 90% bound to human plasma proteins, respectively. Tra is administered at 2 mg once daily, with a mean steady-state C_max_ of approximately 27.1 ng/mL (approximately 0.049 µM) and a plasma protein binding of approximately 97.4%. Ima is administered at 400 mg once daily, with a mean steady-state C_max_ of approximately 2,074 ng/mL (approximately 4.0 µM) and a plasma protein binding of approximately 98% (Verheijen et al., 2017). Considering the high plasma protein binding of these TKIs, their pharmacologically active free (unbound) plasma concentrations are substantially lower than their total concentrations. The concentrations selected in the present study (Snt, 4.11 µM; Tra, 12.21 µM; and Ima, 21.84 µM; all corresponding to the IC_30_ after 72 h in AC16 cells) represent exposure levels that approximate or moderately exceed clinically relevant unbound therapeutic ranges, thereby enabling the assessment of on-target pharmacological effects and potential off-target cardiotoxic mechanisms relevant to patient exposure.

### 2.3 CCK-8 cell viability assays

To measure cardiomyocyte activity, Cell Counting Kit 8 (CCK-8) assays were performed. AC16 cells were seeded into 96-well cell culture plates at a density of 3,500–4,500 cells/well. Snt was used at concentrations of 0.1, 1, 5, 10, and 20 μM; Tra was used at concentrations of 0.5, 1, 5, 10, 20, 50, and 100 μM; and Ima was used at concentrations of 1, 5, 10, 20, and 50 μM. Different drug concentrations were added to the cells, with at least three wells used for each concentration. Control and blank groups were established simultaneously. The treated cells were cultured for 72 h. CCK-8 reagent was added to the wells based on the CCK-8 kit instructions, and the cells were incubated at 37 °C for 1.5 h. The absorbance of each well was recorded at 450 nm using an enzyme reader (Omega, Switzerland), and IC_50_ curves were generated from the resulting data.

### 2.4 Determination of cardiomyocyte injury markers

For biomarker assessment, AC16 cells culture media were collected from each well and centrifuged at 1,000 × g for 10 min at 4 °C, and the clear supernatants were aliquoted and stored at −80 °C until use. This experiment was conducted following the instructions of the N-terminal pro-brain natriuretic peptide (NT-proBNP) and cardiac troponin I (cTnI) enzyme-linked immunosorbent assay (ELISA) kits (ZCiBiO Technology, Shanghai, China; ZC-35149 and ZC-32003). The absorbance at 450 nm was measured via ELISA, and NT-proBNP and cTnI release were calculated. All assay procedures were performed in strict accordance with the manufacturer’s instructions, and sample concentrations were determined using standard curves constructed using serially diluted standards.

### 2.5 Live-cell imaging system to detect cytoplasmic and mitochondrial calcium levels

To measure cytoplasmic and mitochondrial calcium levels, we used the calcium-sensitive fluorescent indicator Fluo-4/AM (Invitrogen, Carlsbad, CA, United States; F14217) and the mitochondrial fluorescent dye Mito-Tracker Red CMXRos (Invitrogen; M46752) for co-staining. AC16 cells were treated with the three TKIs at concentrations of IC_10_, IC_30_, IC_50_, and IC_70_ for 72 h and incubated at 37 °C in the dark for 30 min. The cells were observed using a live-cell imaging system (Sartorius, Göttingen, Germany).

### 2.6 Detection of mitochondrial membrane potential (MMP)

The MMP of AC16 cells was examined using JC-1 Apoptosis Detection Kits. AC16 cells were incubated at 37 °C for 20 min in 100 μL of culture medium mixed with 100 μL of JC-1 staining solution. Changes in red and green fluorescence were observed using a fluorescence microscope to assess MMP activity. Aggregates exhibiting red fluorescence formed at high membrane potential, whereas monomeric forms exhibiting green fluorescence appeared at low membrane potential. A decrease in the red-to-green fluorescence ratio indicates a reduction in membrane potential, which is an early marker of apoptosis.

### 2.7 Determination of the ATP content

We used a bioluminescence assay kit (Enhanced ATP Assay Kit, Beyotime, China; S0027) according to the manufacturer’s instructions and calculated the ATP concentration of AC16 cells using the standard curve. The protein concentration in the lysate was determined using the bicinchoninic acid (BCA) method, and the absolute ATP concentration (μM) was converted to nmol ATP/mg total protein to normalize differences in protein content among samples and enable metabolic activity assessment.

### 2.8 Immunofluorescence

Immunofluorescence imaging was performed in AC16 and hiPSC-CM cells using Mito-Tracker® Red CMXRos and Fluo-4/AM for co-staining. For AC16, the cells were treated with each of the three TKIs at IC_30_ for 72 h. Cytoplasmic calcium was stained using Fluo-4/AM, and mitochondrial calcium was stained using Mito-Tracker Red. The stained cells were plated onto a transparent, TC-treated 48-well microplate for imaging. The cells were stained using the nuclear marker Hoechst and fixed with 4% paraformaldehyde before observation. The cells were observed using a live-cell imaging system. For hiPSC-CMs, after overexpressing SIRT7 in cells using plasmids, the cells were treated with the three TKIs for 72 h. Subsequently, we used Fluo-4/AM and Mito-Tracker Red to stain calcium in cells. The stained cells were seeded onto glass-bottom culture dishes, counterstained with the nuclear marker Hoechst, and fixed with 4% paraformaldehyde before imaging. The cells were observed using a confocal microscope (LSM900, Zeiss, Germany). To evaluate intracellular calcium, a Z-stack was used to observe multiple layers. The Pearson correlation coefficient was used to quantify Ca^2+^ colocalization using the Zeiss Colocalization module in ZEN software, with thresholds determined from single-labeled samples for each channel.

### 2.9 Cellular plasmid transfection

AC16 cells and iPSC-CMs were transfected with a plasmid encoding SIRT7 (pEnter vector with C-terminal Flag and His tags; GenBank: NM_016538). Briefly, 24 h before transfection, cells were seeded in 6-well plates at 60%–80% confluence. The culture medium was replaced 2 h before transfection. For each well, 1 µg of plasmid DNA was diluted in 100 µL of diluent, mixed with 2 µL of transfection reagent, incubated at room temperature for 15–30 min, and added to the cells. Cells were harvested 24–48 h after transfection for downstream assays.

### 2.10 Transmission electron microscopy (TEM)

We collected AC16 cells pellets of approximately 1 × 1 × 2 mm, immersed them in freshly prepared, prechilled 2.5% glutaraldehyde, and fixed at 4 °C overnight. Subsequently, the samples were processed by the Large Instrument Sharing Platform (LISP) at Hebei Medical University and imaged using a transmission electron microscope. For the acquired electron micrographs, we performed a quantitative analysis of mitochondrial–endoplasmic reticulum coupling (MERC), defined as the proportion of the endoplasmic reticulum within 30 nm of mitochondria relative to the total length of the endoplasmic reticulum in the cell.

### 2.11 Proteomic analysis

Three samples were randomly selected from the control group and the group treated with TKIs at the IC_30_ concentration. Following preliminary processing, the samples were sent to Majorbio Co., Ltd. for standardized proteomic analysis of AC16 cells. After the raw data were converted to MGF format using Discoverer, the Mascot engine was used to align them against the UniProt database for peptide/protein identification, and functional annotation was performed using seven major functional databases (e.g., EggNOG, Gene Ontology, and Kyoto Encyclopedia of Genes and Genomes). Proteins with a fold change of >1.5 or <0.67 and a p-value of <0.05 were considered differentially expressed, and no adjustment for multiple comparisons was applied.

### 2.12 Western blotting

Protein expression levels of SIRT7 (Proteintech, Wuhan, China; 12994-1-AP), the mitochondrial calcium uniporter (MCU) (CST, Danvers, MA, United States; 14997), and GAPDH (Proteintech; 10494-1-AP) in the TKI groups were detected using Western blotting analysis. Proteins were extracted from AC16 cells using RIPA lysis buffer containing protease inhibitors (Beyotime, Shanghai, China; #P0013B). Protein concentrations in the resulting lysates were determined using a BCA assay kit (Multi Sciences, Hangzhou, China; PQ0012) and normalized before sample loading. Equal amounts of total protein (30 μg per lane) were loaded onto sodium dodecyl sulfate–polyacrylamide gel electrophoresis (SDS-PAGE) gels, and the Odyssey dual-color infrared system was used to image and analyze the results.

### 2.13 hiPSC-CMs contraction measurement

Cells were plated at 3 × 10^4^ cells per well and maintained under standard culture conditions (37 °C, 5% CO_2_, 95% humidity). The cells began to stabilize after 6 days of resuscitation, and CardioExcyte® 96 (Nanion Technologies, Shanghai, China) was used to monitor hiPSC-CMs contraction by measuring the impedance amplitude under the same environmental conditions. The instrument was set to record for 20 s per scan, and the recorded impedance curve was stored as a trace. Fitting the curve to the mean beat intuitively represents cell contraction. On the day of TKI or SIRT7 overexpression plasmid transfection, to minimize the effects of temperature on the signal, 180 μL of fresh medium was added to each well for 3–4 h for re-equilibration, and the signal was monitored to establish a stable baseline. Subsequently, 20 μL of 10×-supplemented medium was added to each well to achieve the required final concentration. The final drug concentrations were 2 μM Snt, 5 μM Ima, and 3 μM Tra. To reflect pharmacological effects more accurately and ensure comparable cellular baselines within the same experimental group, self-controlled changes in contraction before and after treatment were assessed as an indicator of drug toxicity.

### 2.14 Statistical analyses

All data are expressed as mean ± standard error of the mean. Normality was assessed using the Shapiro–Wilk test, and homogeneity of variances was evaluated using the Levene’s test. For comparisons between two groups, an unpaired Student’s t-test was used for normally distributed data with equal variances; otherwise, the Mann–Whitney *U* test was used. For multiple-group comparisons, one-way analysis of variance was used when the assumptions of normality and homogeneity of variances were met, followed by Tukey’s honestly significant difference test for all pairwise comparisons, with *P*-values adjusted to control the family-wise error rate. For non-normally distributed data, the Kruskal–Wallis test was used, followed by Dunn’s test for multiple comparisons. All statistical analyses were performed using GraphPad Prism 8 (GraphPad Software, San Diego, CA, United States). A significance level (α) of 0.05 was established, with *P* < 0.05 deemed statistically significant. Fluorescence images were quantitatively analyzed using ImageJ software (National Institutes of Health, United States) and the colocalization module of ZEN software (Zeiss, Germany). Each independent experiment was performed at least three times.

## 3 Results

### 3.1 Three TKIs induce cardiomyocyte injury in AC16 cells by regulating mitochondrial Ca^2+^overload

In AC16 cells, Snt (0.1, 1, 5, 10, and 20 μM), Tra (0.5, 1, 5, 10, 20, 50, and 100 μM), and Ima (1, 5, 10, 20, and 50 μM) incubation for 72 h resulted in concentration-dependent cytotoxicity, with IC_50_ values of 6.62 μM, 28.94 μM, and 18.44 μM, respectively (Figures 1A–C). Consistent with the *in vitro* cytotoxicity classification specified in ISO 10993–5 (ISO nOfS, 1994), concentrations corresponding to no toxicity (IC_10_), low toxicity (IC_30_), and moderate toxicity (IC_50_ and IC_70_) were selected for subsequent experiments. At IC_30_, IC_50_, and IC_70_, all three TKIs substantially increased the levels of cardiomyocyte injury markers NT-proBNP and cTnI in a concentration-dependent manner (Figures 1D,E). Normal contraction of cardiac muscle cells depends on ATP supply and the ECC, a process regulated by various kinases, such as protein kinase II, phosphoinositide 3-kinase (PI3K), and AMP-activated protein kinase. Several TKIs can interfere with the function of these kinases (Kerkela et al., 2009; Lu et al., 2012; Mooney et al., 2015). Thus, irrespective of their initial targets, TKIs may ultimately disrupt Ca^2+^ homeostasis in cardiomyocytes through these kinase pathways, leading to contractile dysfunction. The three TKIs investigated in this study caused cytoplasmic Ca^2+^ overload in AC16 cells at IC_10_, IC_30_, IC_50_, and IC_70_ (Figures 1F,G).

**FIGURE 1:**
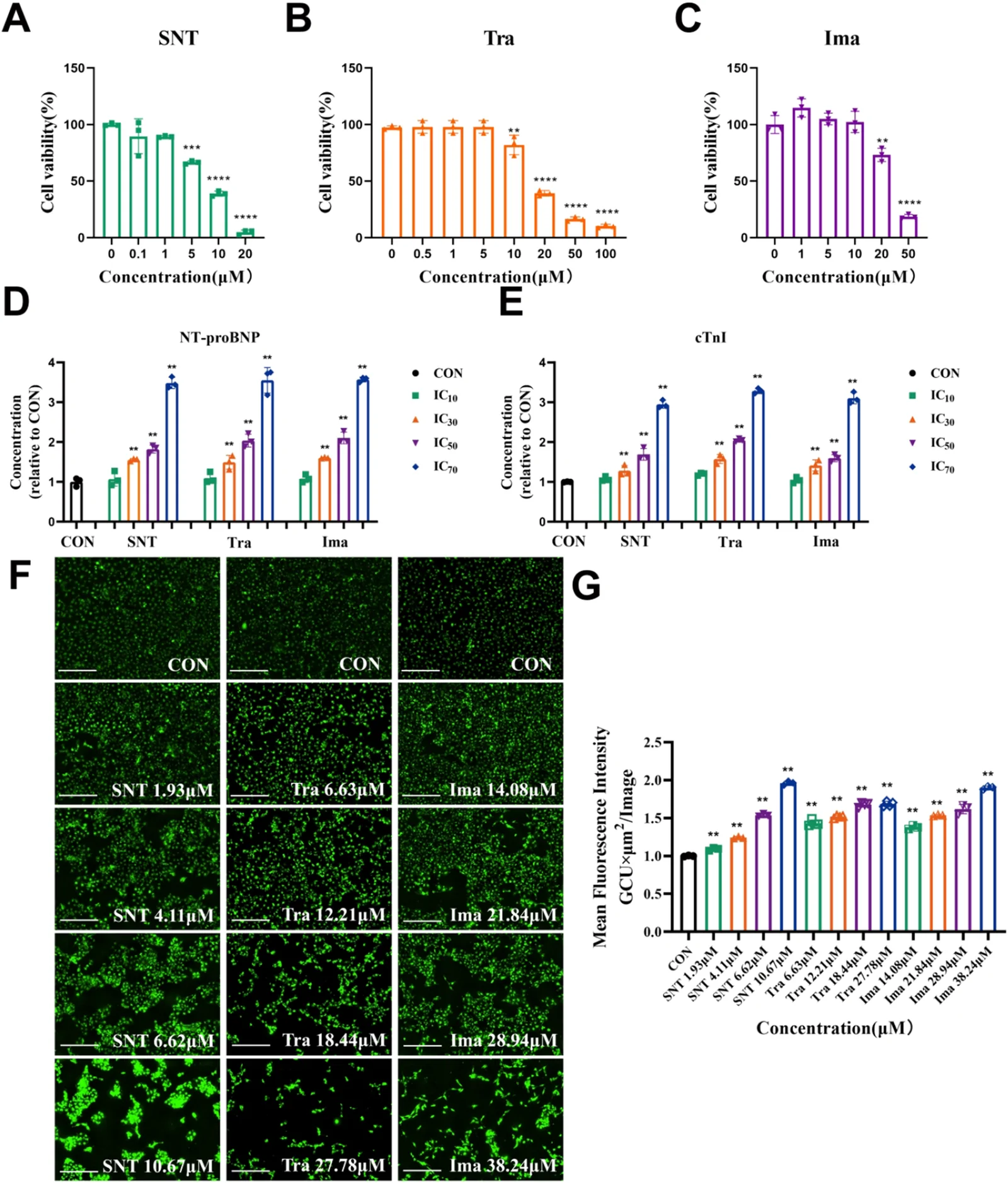
TKIs induce AC16 cell injury and cytosolic Ca^2+^ overload. **(A–C)** IC_50_ curves for AC16 cells treated with Snt, Tra, and Ima for 72 h. **(D,E)** At IC_10_, IC_30_, IC_50_, and IC_70_, TKIs cause a concentration-dependent increase in NT-proBNP and cTnI in AC16 cells (n = 3, compared with the control group, ^**^ *P* < 0.01). **(F)** A representative image of TKI-induced cytosolic Ca^2+^ overload at IC_10_, IC_30_, IC_50_, and IC_70_, as detected by live-cell imaging (scale: 400 μm). **(G)** TKI-induced cytosolic Ca^2+^ overload in AC16 cells (n = 3, compared with the control group, ^**^ *P* < 0.01). cTnI, cardiac troponin I; Ima, imatinib; NT-proBNP, N-terminal pro-brain natriuretic peptide; Snt, sunitinib; Tra, trametinib; TKIs, tyrosine kinase inhibitors.

Ca^2+^ is essential for mitochondrial function. It helps activate the respiratory chain and ATP production and regulates ECC by buffering cytoplasmic Ca^2+^ concentrations (Gustafsson and Gottlieb, 2008; Kim et al., 2018). To investigate the effects of TKIs on calcium homeostasis between mitochondria and the endoplasmic reticulum, we measured mitochondrial calcium levels. Considering that TKIs increased the levels of the cardiomyocyte injury markers NT-proBNP and cTnI as well as cytoplasmic Ca^2+^ overload across four concentrations, we selected the lowest TKIs concentration (IC_30_) at which significant differences were observed in the subsequent experiments. We used the mitochondrial fluorescent probe Mito-Tracker Red to visualize the mitochondria and Fluo-4 to detect Ca^2+^ levels. Mitochondrial Ca^2+^ levels can be quantified by the intensity of green fluorescence at colocalization sites between the red and green signals. As shown in Figure 2A, green fluorescence intensity in the colocalized regions increased significantly following TKI treatment, indicating a marked increase in mitochondrial Ca^2+^ levels. Additionally, the red-green fluorescence image in the rightmost section demonstrates a high degree of colocalization between the red and green signals. The shift from red-to-green fluorescence in the JC-1 experiment clearly indicates a decrease in MMP in the TKI-treated groups (Figures 2B–D). The TKI-treated group had significantly decreased ATP levels, indicating impaired mitochondrial function (Figure 2C).

**FIGURE 2:**
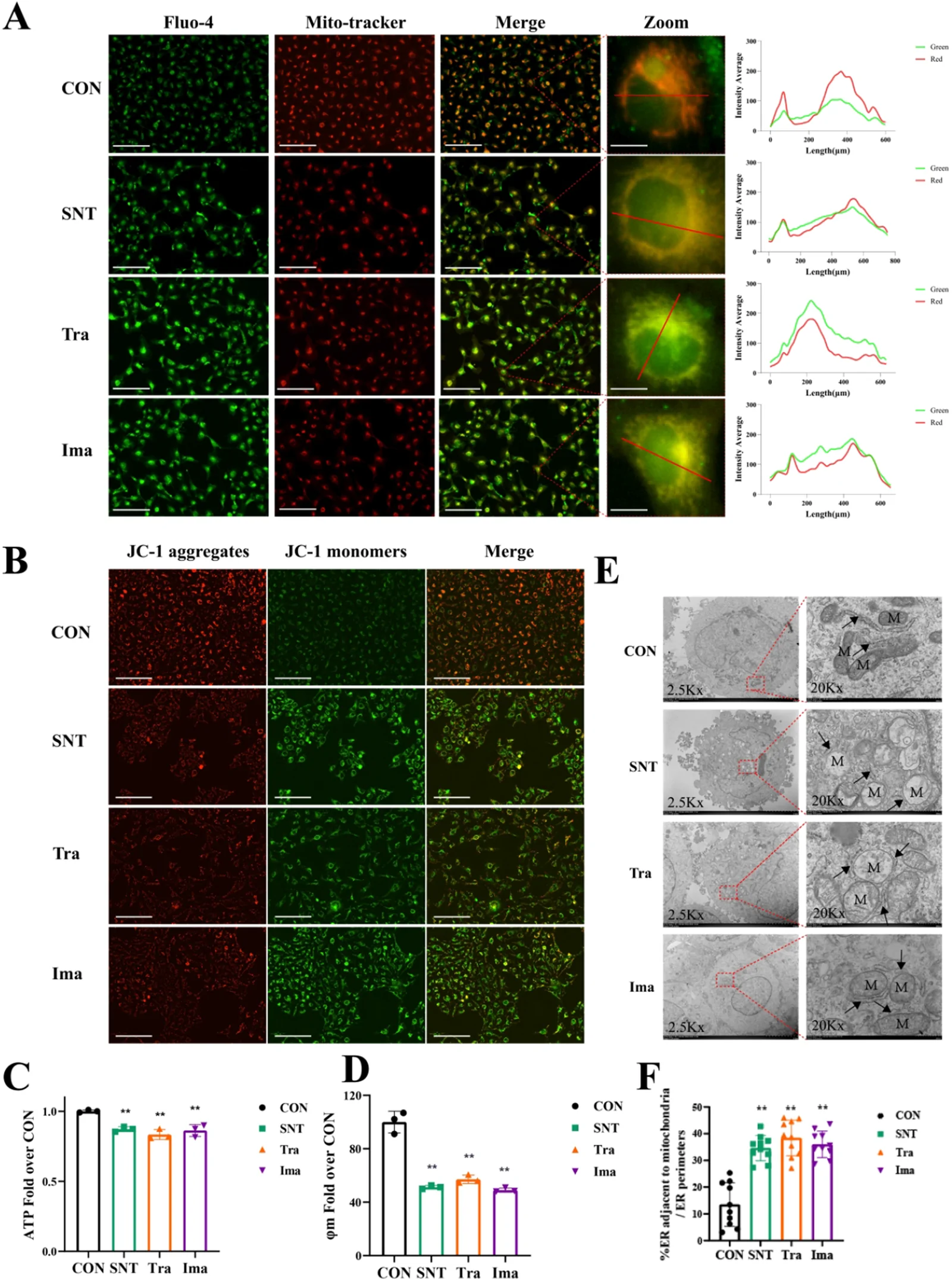
TKIs impair mitochondrial function and morphology in AC16 cells. **(A)** Effect of TKIs on mitochondrial Ca^2+^ content of AC16 cells detected by live-cell imaging; scale: 200 μm. **(B)** Effect of TKIs on Δφm of AC16 cells detected by live-cell imaging; scale: 200 μm. **(C)** TKI-induced changes in mitochondrial ATP content. **(D)** Δφm induced by TKIs, expressed as green/red fluorescence intensity ratio (n = 3, compared with the control group, ^**^ *P* < 0.01). **(E)** Transmission electron micrograph of AC16 cells showing the effect of TKIs on mitochondrial morphology; the arrow represents the endoplasmic reticulum and M represents mitochondria; scale bar: 2.5 Kx, 50 μm; 20 Kx, 500 nm. **(F)** MERC formation was quantified. TKIs significantly increased MERC% in AC16 cells (n = 10, compared with the control group, ^**^ *P* < 0.01). ATP, adenosine triphosphate; M, mitochondria; MERC, mitochondrial–endoplasmic reticulum coupling; TKIs, tyrosine kinase inhibitors.

We examined the effects of TKIs on mitochondrial morphology and MERC. To quantify the MERC, we used image segmentation to measure the proportion of endoplasmic reticulum length within 30 nm of mitochondria relative to the total endoplasmic reticulum length in the cell, characterizing the spatiotemporal dynamics of mitochondrial–endoplasmic reticulum contacts. TEM revealed that TKIs induce morphological abnormalities, such as mitochondrial swelling and vacuolization, and substantially reduce the average distance between the endoplasmic reticulum and mitochondria, resulting in a marked increase in MERCs (Figures 2E,F).

### 3.2 SIRT7 upregulation protects AC16 cardiomyocytes from TKI-induced mitochondrial Ca^2+^ overload

To further elucidate the common mechanisms underlying TKI-induced cardiotoxicity, we used proteomics to detect changes in protein expression following TKI treatment and validate their functions by modulating protein expression. As shown in the proteomics Venn diagrams and volcano plots in Figures 3A–E, compared with the control group, the Snt group had 355 differentially expressed proteins, including 226 upregulated and 129 downregulated proteins; the Tra group had 933 differentially expressed proteins, including 649 upregulated and 284 downregulated proteins; and the Ima group had 1,220 differentially expressed proteins, including 989 upregulated and 231 downregulated proteins. There were 67 common upregulated and 13 downregulated proteins across the three groups. The heatmap illustrates the overall trends in protein expression for 1,870 proteins in the control and TKI groups (Figure 3F). Among the 13 proteins consistently downregulated across all samples, SIRT7 was identified as a key candidate because of its established roles in regulating endoplasmic reticulum stress, energy deprivation, hypoxia, DNA damage, and mitochondrial function, all of which are closely associated with the TKI-induced cardiotoxicity phenotype observed in this study. Therefore, subsequent experiments were focused on assessing whether SIRT7 upregulation protects against TKI-induced myocardial injury.

**FIGURE 3:**
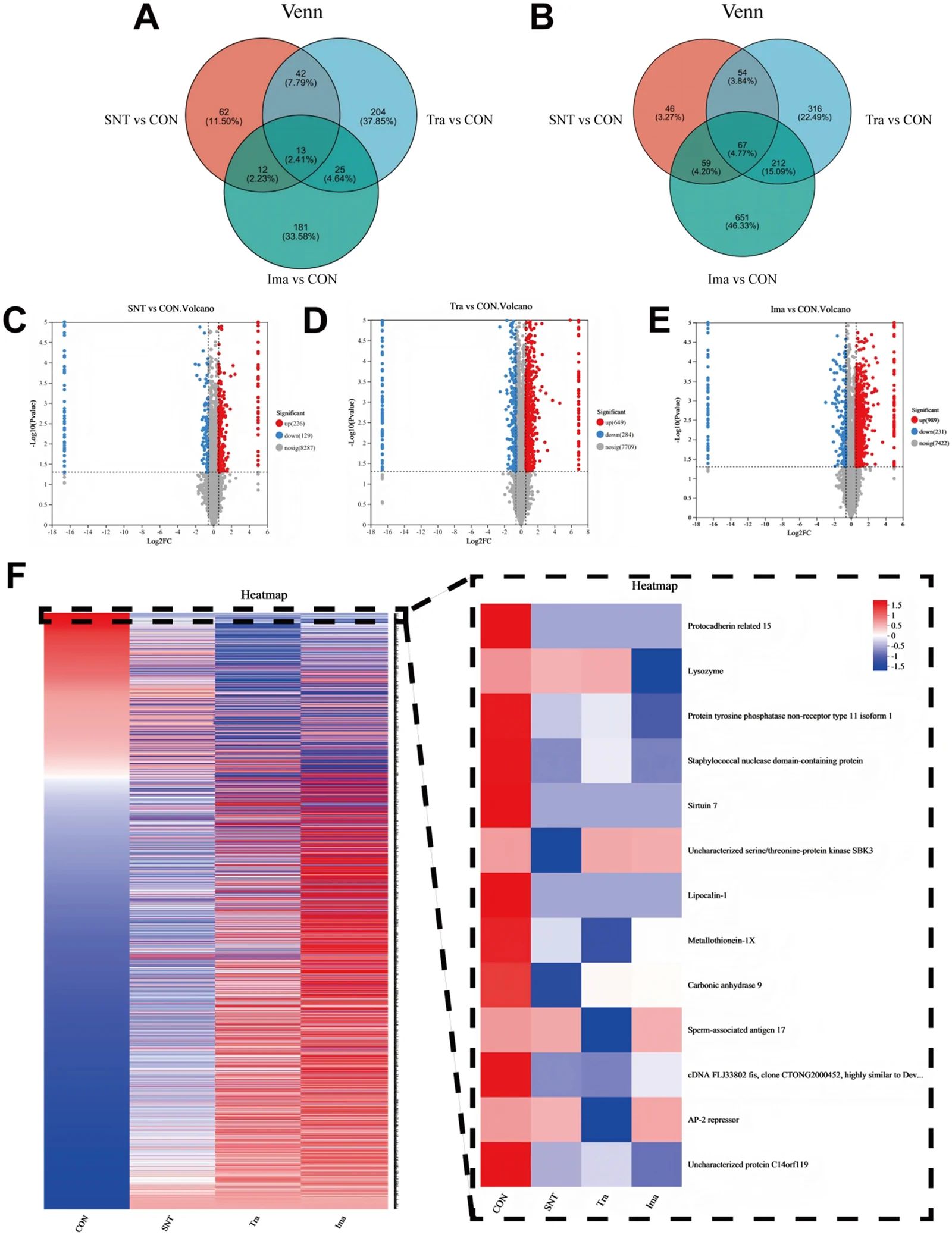
Effects of TKIs on protein expression changes in AC16 cells. **(A,B)** Venn diagram of TKI-induced downregulation and upregulation of proteins in AC16 cells. **(C–E)** Volcano plots showing TKI-induced changes in AC16 protein expression. **(F)** Heatmap of TKI-induced AC16 protein expression changes. Each column in the figure represents a sample. Each line represents a protein. TKIs, tyrosine kinase inhibitors.

To validate SIRT7 function, we overexpressed SIRT7 in AC16 cells via plasmid transfection. As illustrated in the Western blot results in Figures 4A,B, transfection was successful. Results from the CCK-8 and ELISA assays revealed that SIRT7 overexpression significantly protected against reduced AC16 cell viability and elevated cardiac injury markers NT-proBNP and cTnI induced by TKIs (Figures 4C–E). Furthermore, SIRT7 overexpression effectively alleviated TKI-induced cytoplasmic Ca^2+^ (Figures 4F,G) and mitochondrial Ca^2+^ overload (Figure 5A). Mitochondrial function was substantially improved, as evidenced by the reversal of the decline in ATP levels and a reduction in MMP (Figures 5B–D). Furthermore, TEM revealed that SIRT7 overexpression alleviated TKI-induced mitochondrial morphological abnormalities, such as swelling and vacuolization, and reduced the excessively elevated proportion of functional MERC (Figures 5E,F). In summary, SIRT7 overexpression provides significant protection against TKI-induced cardiomyocyte damage, calcium homeostasis disruption, and mitochondrial dysfunction.

**FIGURE 4:**
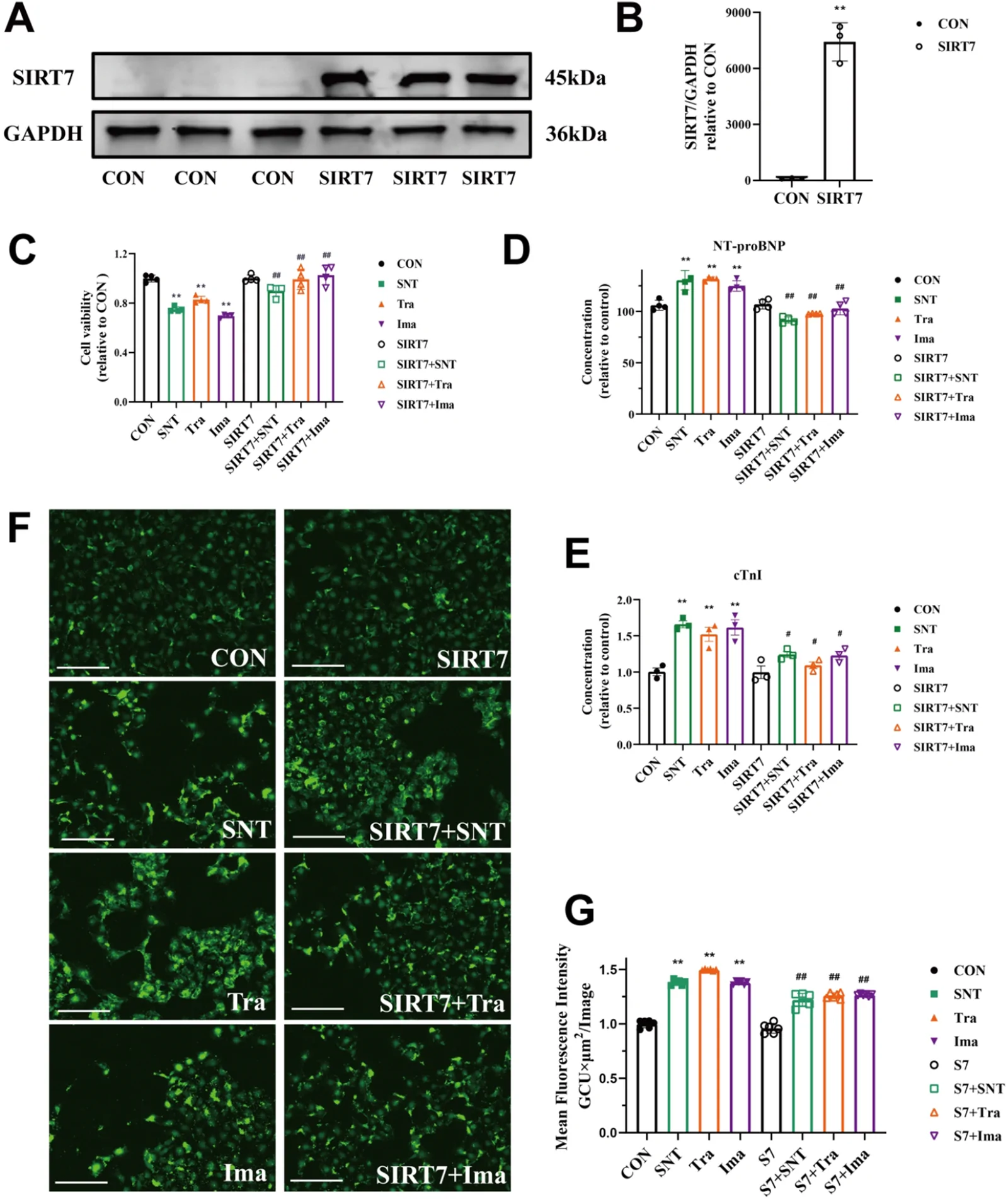
SIRT7 overexpression protects against TKI-induced AC16 cell injury and cytosolic Ca^2+^ overload. **(A)** SIRT7 protein expression was detected using Western blotting. **(B)** SIRT7 overexpression (n = 3, compared with the control group, ^**^ *P* < 0.01). **(C)** SIRT7 upregulation protects against TKI-induced cell viability decline (n = 4, compared with the control group, ^**^ *P* < 0.01; compared with TKI treatment alone, ^##^ *P* < 0.01). **(D,E)** SIRT7 upregulation protects against TKI-induced elevation of the myocardial injury markers NT-proBNP and cTnI (n = 3, compared with the control group, ^**^ *P* < 0.01; compared with TKI treatment alone, ^#^ *P* < 0.05, ^##^ *P* < 0.01). **(F)** SIRT7 overexpression protects against TKI-induced cytosolic calcium overload; scale: 200 μm. **(G)** SIRT7 overexpression attenuates TKI-induced cytosolic calcium overload (n = 6, compared with the control group, ^**^ *P* < 0.01; compared with the TKI treatment group, ^##^ *P* < 0.01). cTnI, cardiac troponin I; NT-proBNP, N-terminal pro-brain natriuretic peptide; SIRT7, sirtuin 7; TKIs, tyrosine kinase inhibitors.

**FIGURE 5:**
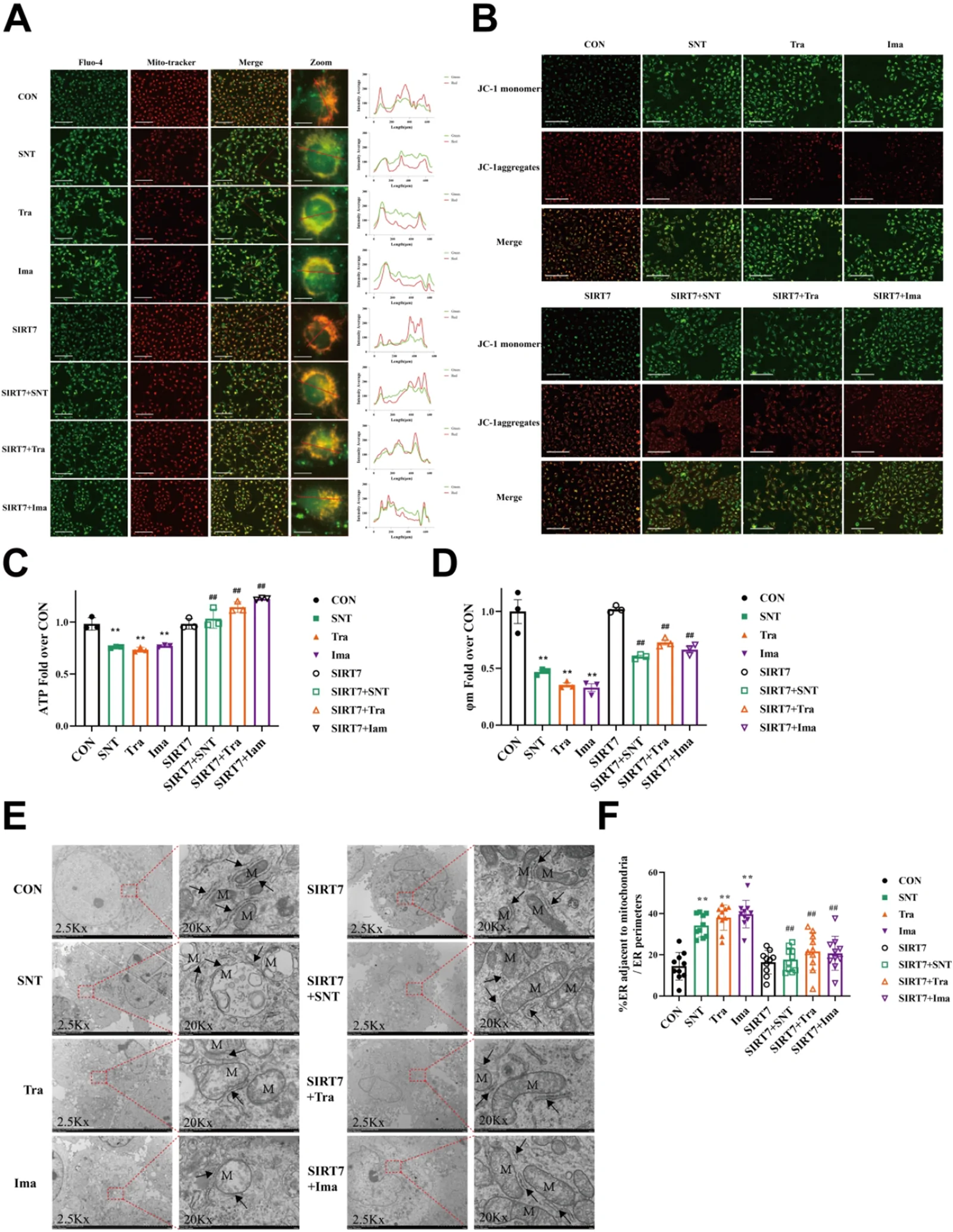
SIRT7 upregulation rescues TKI-induced declines in ATP production and Δφm and preserves endoplasmic reticulum–mitochondria functional coupling in AC16 cells. **(A)** SIRT7 upregulation mitigates TKI-induced mitochondrial Ca^2+^ overload. Compared with the control group, the three tested TKIs caused AC16 mitochondrial Ca^2+^ overload, which was reversed by SIRT7 upregulation; scale: 200 μm. **(B)** Effect of SIRT7 upregulation on Δφm of AC16 cells (scale: 200 μm). **(C)** SIRT7 upregulation induced changes in mitochondrial ATP content. **(D)** Δφm induced by SIRT7 upregulation, expressed as green/red fluorescence intensity ratio (n = 3, compared with the control group, ^**^ *P* < 0.01; compared with TKI treatment, ^##^ *P* < 0.01). **(E)** Transmission electron micrograph of AC16 cells showing the effect of SIRT7 upregulation on mitochondrial morphology, in which the arrow represents the endoplasmic reticulum and M represents mitochondria; scale bar: 2.5 Kx, 50 μm; 20 Kx, 500 nm. **(F)** MERC formation was quantified (n = 10, compared with the control group, ^**^ *P* < 0.01; compared with TKI-treated alone, ^##^ *P* < 0.01). ATP, adenosine triphosphate; M, mitochondria; MERC, mitochondrial–endoplasmic reticulum coupling; SIRT7, sirtuin 7; TKIs, tyrosine kinase inhibitors.

### 3.3 MCU is a key target for SIRT7 in protecting against TKI-induced cardiomyocyte injury

Interactions between mitochondria and the endoplasmic reticulum, particularly Ca^2+^ transport, are critical for regulating cell survival and death (Rimessi et al., 2008; Williams et al., 2015). MMP drives ATP synthesis and provides the driving force for Ca^2+^ to enter the mitochondrial matrix along its electrochemical gradient (Morciano et al., 2015). This process is primarily mediated by the MCU complex, which serves as the main pathway for cytoplasmic Ca^2+^ to enter the mitochondria. Further analysis of the proteomics results revealed that TKIs reduced SIRT7 expression and increased MCU expression in the inner mitochondrial membrane (Figures 6A,B). Thus, the protective effect of SIRT7 may be mediated by MCU regulation.

**FIGURE 6:**
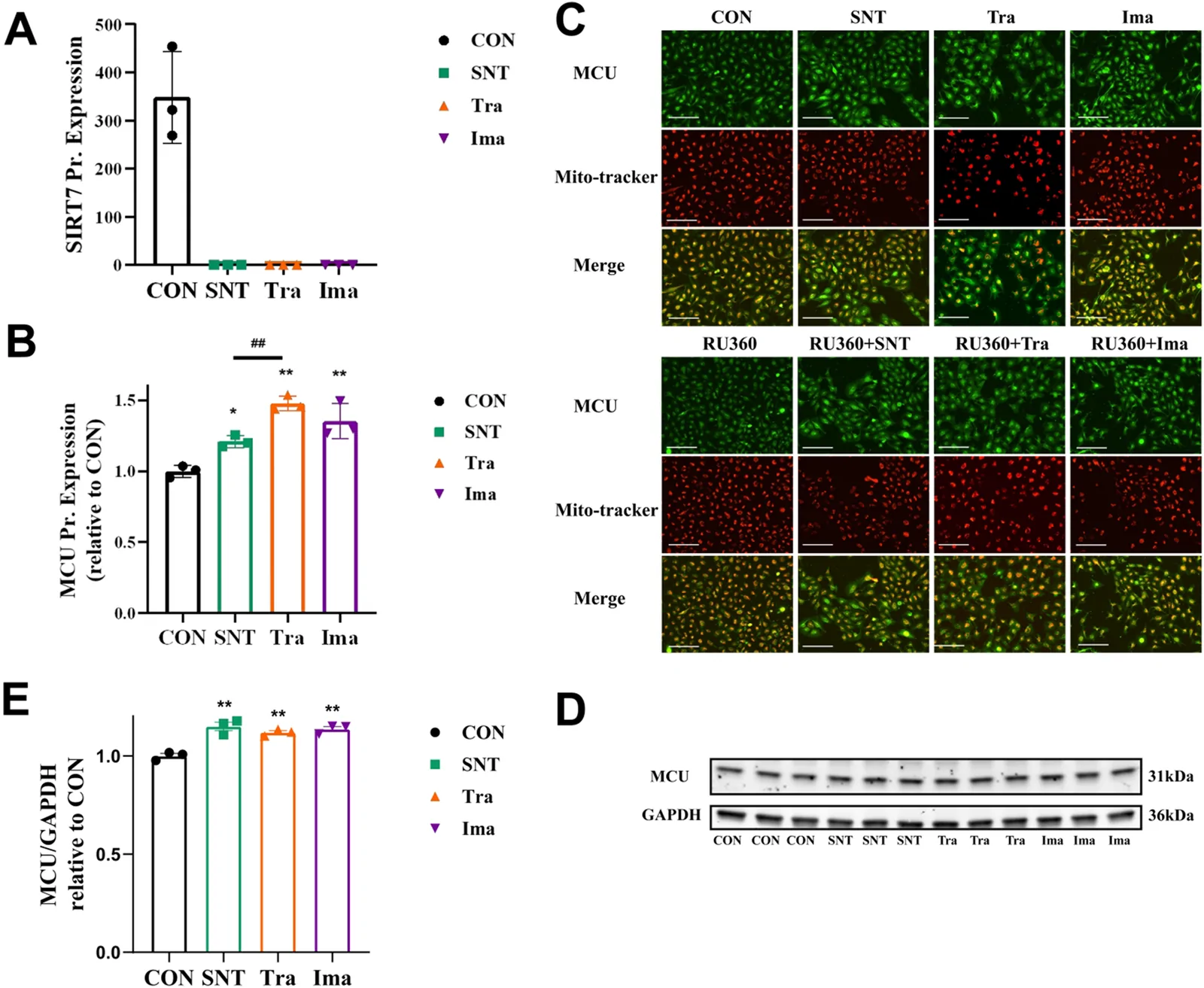
TKIs activate the MCU, leading to mitochondrial Ca^2+^ overload in AC16 cells. **(A)** The tested TKIs significantly reduced SIRT7 expression. **(B)** TKIs significantly enhanced MCU expression (n = 3, compared with the control group, *P < 0.05, **P < 0.01; compared with the Snt group, ^##^ *P* < 0.01). **(C)** Green fluorescence intensity represents the MCU activity. Before TKI administration, preincubation of RU360 at 5 μM for 2 h effectively inhibited MCU functional enhancement; scale, 200 μm. **(D,E)** TKIs increased AC16 MCU protein expression (n = 3, compared with the control group, **P < 0.01). MCU, mitochondrial calcium uniporter; SIRT7, sirtuin 7; Snt, sunitinib; TKIs, tyrosine kinase inhibitors.

We performed MCU function and expression assays to test the above hypothesis. Ru360, a selective inhibitor of mitochondrial calcium uptake, effectively blocks Ca^2+^ uptake into mitochondria and emits green fluorescence. Immunofluorescence analysis revealed that TKI treatment significantly enhanced MCU activity, as indicated by increased green fluorescence, and Ru360 inhibited this effect (Figure 6C). Western blotting confirmed that TKIs also increased MCU protein expression (Figures 6D,E). Notably, following SIRT7 overexpression, the TKI-induced increase in MCU protein expression (Figures 7A,B) and enhanced activity (Figure 7C) were significantly reversed. These results suggest that SIRT7 protects cells from TKI-induced calcium overload and mitochondrial damage by inhibiting excessive MCU activation.

**FIGURE 7:**
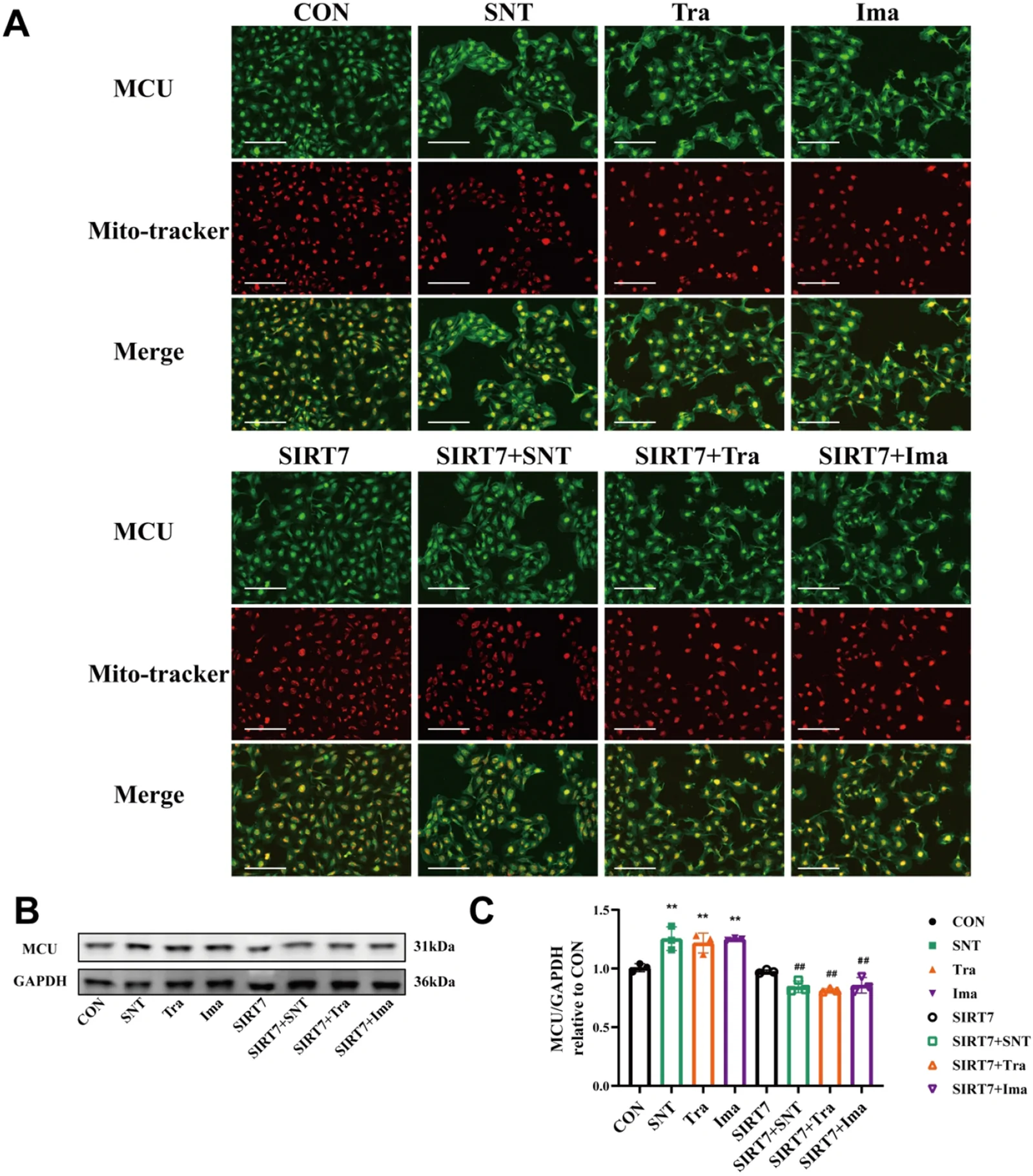
SIRT7 Upregulation in AC16 cells counteracts TKI-induced MCU activation and mitochondrial Ca^2+^ overload. **(A)** Green fluorescence intensity represents the MCU activity. SIRT7 upregulation effectively inhibits MCU functional enhancement before TKI administration; scale: 200 μm. **(B,C)** SIRT7 upregulation counteracts the increase in MCU protein expression (n = 3, compared with the control group, **P < 0.01; compared with TKI treatment, ^##^ *P* < 0.01). MCU, mitochondrial calcium uniporter; SIRT7, sirtuin 7; TKIs, tyrosine kinase inhibitors.

### 3.4 SIRT7 upregulation protects against TKI-induced mitochondrial Ca^2+^ overload and contractile dysfunction in hiPSC-CMs

To further validate the protective effect of SIRT7 upregulation against TKI-induced myocardial contractile dysfunction, we examined the effects of TKI activation on mitochondrial Ca^2+^ homeostasis and contractile inhibition in hiPSC-CMs. Snt, Ima, and Tra substantially reduced the contraction amplitude in hiPSC-CMs. We overexpressed SIRT7 in hiPSC-CMs via plasmid transfection and found that the reduction in cell contractility induced by the three TKIs was reversed (Figures 8A–F). Immunofluorescence results showed that TKI treatment significantly increased MCU activity in hiPSC-CMs and suppressed SIRT7 expression (Figure 9). In addition, SIRT7 overexpression significantly protected against TKI-induced mitochondrial Ca^2+^ overload (Figure 8G). These results indicate that SIRT7 upregulation protects hiPSC-CMs from TKI-induced MCU activation and mitochondrial Ca^2+^ overload, preventing contraction inhibition.

**FIGURE 8:**
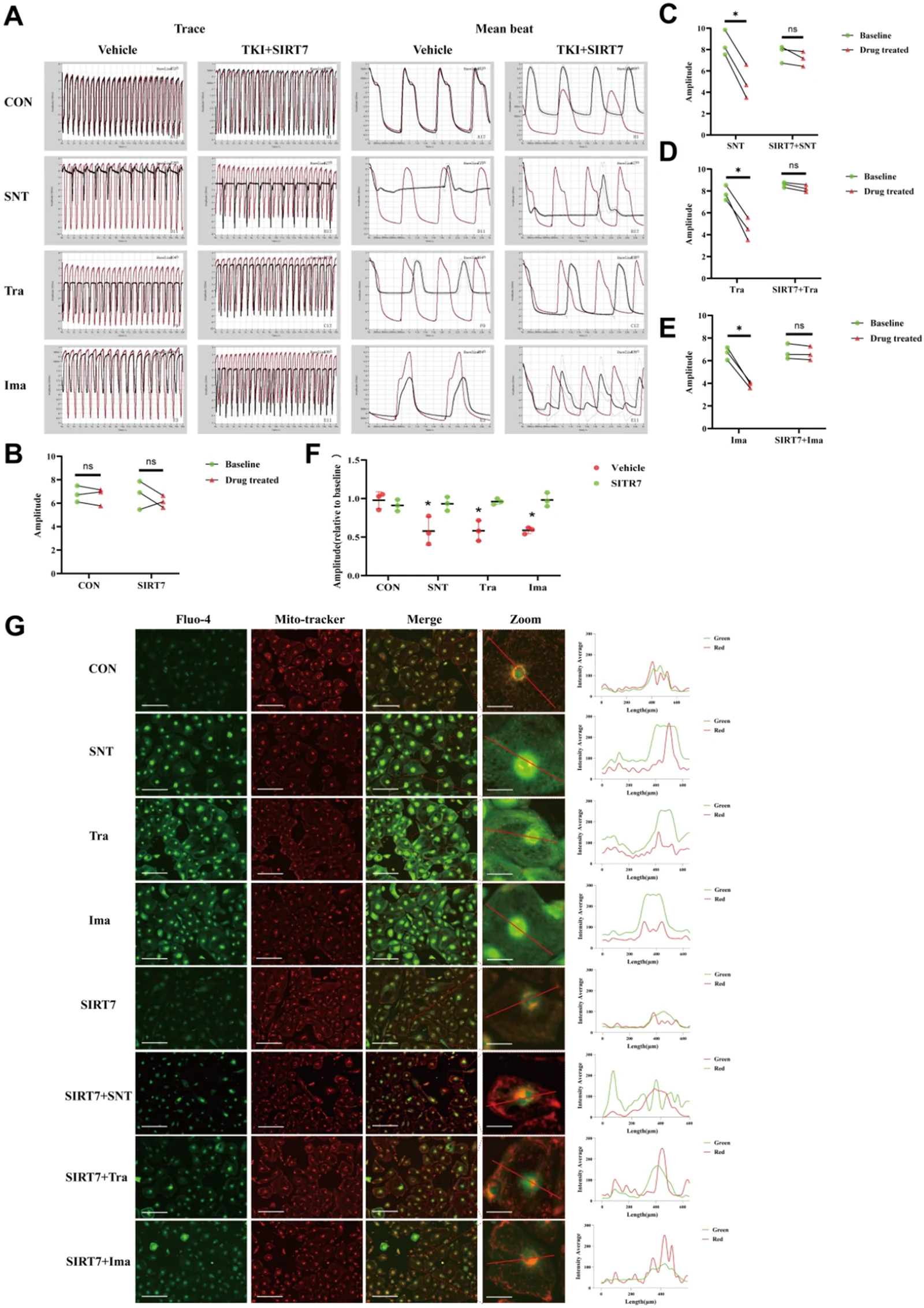
SIRT7 upregulation rescues TKI-induced declines in hiPSC-CMs. **(A–F)** TKIs inhibited the mean beating frequency at 72 h, and SIRT7 pretreatment protected against the impedance amplitude reduction induced by TKIs (n > 4, compared with the control group, ^**^ *P* < 0.01; compared with the TKI treatment alone, ^##^ *P* < 0.01). **(G)** SIRT7 upregulation mitigates TKI-induced mitochondrial Ca^2+^ overload. Compared with the control group, the three TKIs caused mitochondrial Ca^2+^ overload in hiPSC-CMs, which was reversed by SIRT7 upregulation; scale: 200 μm. hiPSC-CMs, human induced pluripotent stem cell–derived cardiomyocytes; SIRT7, sirtuin 7; TKIs, tyrosine kinase inhibitors.

**FIGURE 9:**
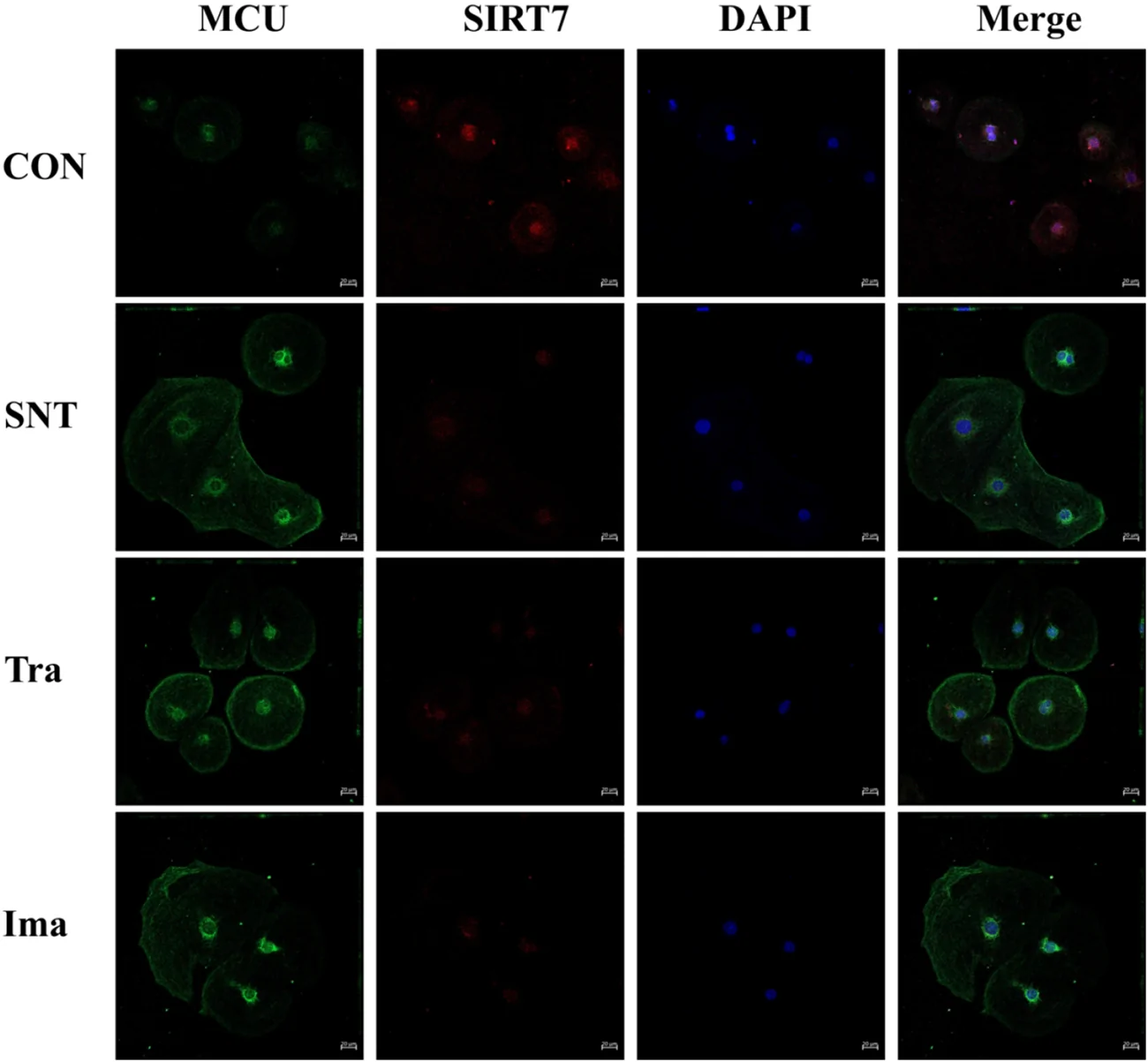
Treatment with TKIs enhanced MCU activity and suppressed SIRT7 expression in hiPSC-CMs. Green fluorescence intensity represents the MCU activity. Red fluorescence intensity represents the SIRT7 activity. TKIs significantly reduced SIRT7 expression and increased MCU expression; scale: 20 μm. MCU, mitochondrial calcium uniporter. MCU, mitochondrial calcium uniporter; SIRT7, sirtuin 7; TKIs, tyrosine kinase inhibitors.

## 4 Discussion

TKIs are a highly effective class of anticancer drugs. They inhibit phosphorylation in downstream signaling pathways by blocking the binding of intracellular kinases to ATP, counteracting excessive tyrosine kinase activation and suppressing tumor cell proliferation. They have been widely used in the treatment of various malignant tumors and have substantially improved patient survival rates. However, TKIs are associated with a wide range of adverse reactions, of which cardiac toxicity is the most severe. This primarily manifests as left ventricular systolic dysfunction, heart failure, and electrocardiogram-detected QT interval prolongation and arrhythmias (Gharwan and Groninger, 2016; Chu et al., 2007). Cardiotoxicity associated with TKIs may be caused by on- and off-target effects (Force and Kolaja, 2011; Truitt et al., 2018). These mainly include interfering with myocardial ion channels (Li et al., 2023), kinase signaling pathways, and energy metabolism (Chen Y.-T. et al., 2023); affecting the cardiac conduction system (Singh et al., 2019), mitochondrial function, and calcium regulation; and inducing oxidative stress and inflammatory responses (Pfister et al., 2014; Wang et al., 2023), resulting in myocardial cell damage or even apoptosis (Grabowska et al., 2021; Monogiou Belik et al., 2024), which in turn impairs cardiac function. We found that SIRT7 is a common target of cardiotoxicity among the three tested TKIs; SIRT7 upregulation to counteract MCU activation significantly mitigates TKI-induced damage to endoplasmic reticulum–mitochondrial interactions and protects against myocardial injury.

The mechanisms of cardiotoxicity may vary among different TKIs, depending on their specific targets and pharmacological properties. Snt disrupts calcium regulation in cardiomyocytes, thereby impairing cardiac contractility (Li et al., 2019). It also induces cardiac hypertrophy via the endothelin pathway (Sourdon et al., 2021; Sourdon et al., 2017). Osimertinib-induced PDK4 upregulation may be a major cause of cardiomyopathy (Deng et al., 2025). TKIs have been reported to potentially cause myocardial injury through a common mechanism. Inhibition of WNK1 kinase activity is a common mechanism underlying arrhythmias induced by nilotinib, vandetanib, and mobocetinib (Wu et al., 2024). Our preliminary findings showed that Snt, Ima, and Tra can cause calcium regulation disorders and contractile dysfunction in cardiomyocytes by inhibiting PI3K kinase activity (Gao et al., 2025). Based on previous findings, this study investigated whether single-target and multitarget drugs cause myocardial injury through abnormal calcium regulation and aimed to identify the underlying mechanisms. We found that Snt, Ima, and Tra induce cytosolic calcium overload and activate the MCU at the mitochondria-associated membrane, leading to mitochondrial calcium overload, which in turn causes cardiomyocyte injury and contraction inhibition.

Sirtuins are a family of NAD^+^-dependent deacetylases in the nucleus (e.g., sirtuin 1, sirtuin 6, and SIRT7), cytoplasm (sirtuin 2), and mitochondria (sirtuin 3–5). They contribute to aging and disease processes by regulating gene expression, metabolic homeostasis, and stress responses (Shen et al., 2024). Proteomic analysis revealed significantly downregulated SIRT7 expression levels. Because sirtuin function is primarily dependent on the NAD^+^/NADH ratio, this protein family can serve as a sensor of cellular energy status (Higurashi et al., 2025). This may be particularly important in mitochondria, as they harbor numerous acetylated proteins and contain high levels of NAD^+^ and NADH. SIRT7 maintains nucleolar function by deacetylating ribosomal RNA transcription regulators (e.g., RNA polymerase I) and indirectly influences mitochondrial protein synthesis and energy metabolism (Ding et al., 2025). Resveratrol protects cardiomyocytes from oxidative stress–induced apoptosis by activating SIRT1, 3, 4, and 7 (Yu et al., 2009). In mice, SIRT7 deficiency has been reported to induce multisystemic mitochondrial dysfunction (Ryu et al., 2014). Consistent with our experimental results, all three tested TKIs caused mitochondrial dysfunction, including decreased MMP and reduced ATP levels; SIRT7 upregulation exerted a significant protective effect.

Mitochondrial Ca^2+^ uptake has long been recognized to be involved in the upregulation of oxidative metabolism in various cells and tissues. In the heart, it supports steady-state cardiac metabolism and function under basal conditions and is particularly important under stress conditions (Boyman et al., 2021; Pan et al., 2013). Mitochondrial Ca^2+^ uptake is primarily mediated by the MCU. The MCU is a Ca^2+^ channel composed of four MCU subunits, with its pore structure supported by the essential MCU regulator (EMRE). Because of the strong driving force provided by the inner MMP, the MCU-EMRE pore alone can mediate mitochondrial Ca^2+^ influx even at the lowest cytosolic free calcium concentration ([Ca^2+^]c) (Tsai et al., 2016; Yamamoto et al., 2016). However, both MCU and EMRE bind to homodimers or heterodimers composed of the MICU1 and MICU2 or MICU3 proteins; these dimers inhibit mitochondrial Ca^2+^ entry at resting [Ca^2+^]c levels and mediate the synergistic activation of Ca^2+^ uptake when [Ca^2+^]c increases (Csor et al., 2013; Kamer et al., 2017). Excessive mitochondrial Ca^2+^ uptake leads to mitochondrial membrane permeabilization and cell death (Bernardi and Rasola, 2007; Hajnóczky et al., 2000). Studies have shown that myocardial ischemia leads to mitochondrial Ca^2+^ overload and cell death; in heart failure, excessive and insufficient mitochondrial Ca^2+^ uptake have been reported to contribute to pathological changes (Liu et al., 2021; Santulli et al., 2015). We found that treatment with the tested TKIs increased MCU protein expression in AC16 cells and hiPSC-CMs; however, a significant difference was observed in the extent of the increase between Snt and Tra, which may be due to differences in the number of targets they act upon. SIRT7 overexpression reverses MCU activation and expression levels, suggesting that the protective effect of SIRT7 against TKI-induced cardiomyocyte injury may depend on its regulation of the balance between mitochondrial calcium uptake and release, providing a new mechanistic basis for TKI-induced cardiotoxicity. Plasma SIRT7 levels are lower in patients with hypertension than in those without hypertension, and SIRT7 is strongly associated with hypertension-induced cardiac hypertrophy and heart failure (Liu et al., 2024). Therefore, SIRT7 expression levels may serve as a biomarker for predicting TKI-induced cardiotoxicity.

In summary, this study is the first to demonstrate the protective role of SIRT7 against TKI-induced cardiomyocyte injury. This study provides theoretical support for the clinical monitoring, prevention, and treatment of cardiac dysfunction associated with TKI-class drugs and advances the field of oncocardiology. Future studies should validate the molecular mechanism by which SIRT7 mediates MCU activity in animal models, followed by an investigation of the association between SIRT7 expression and TKI-induced cardiotoxicity in clinical samples.

### 4.1 Limitations

The present study has several limitations. First, although the use of hiPSC-CMs enhances the translational relevance of our findings, all experiments were conducted *in vitro*. Therefore, *in vivo* validation in animal models is required to verify the cardioprotective effects of SIRT7 upregulation and evaluate its effects on overall cardiac function under clinically relevant TKI dosing regimens. Second, although our data indicate that SIRT7 overexpression reduces MCU protein expression and activity, the underlying molecular mechanisms remain unclear. Specifically, whether SIRT7 directly deacetylates MCU or its regulatory subunits, such as MICU1, MICU2, or EMRE, or indirectly suppresses MCU transcription through epigenetic regulation or transcription factor modulation remains to be determined. Therefore, further mechanistic studies are necessary. Third, although we selected three TKIs with distinct primary targets to represent both multitarget and single-target agents, these compounds may not fully represent the diversity of clinically approved TKIs. Therefore, the generalizability of our findings to other TKIs should be interpreted with caution. Fourth, the relationship between endogenous SIRT7 expression and the clinical incidence of TKI-induced cardiotoxicity in patients with cancer remains unexplored. Prospective clinical cohort studies are necessary to determine whether circulating or myocardial SIRT7 levels could serve as predictive biomarkers for the early detection of cardiotoxicity. Addressing these limitations in future studies will help establish the therapeutic and translational potential of targeting the SIRT7–MCU axis as a cardioprotective strategy in patients receiving TKI-based anticancer therapy.

### 4.2 Conclusion

This study found that three TKIs—Snt, Tra, and Ima—targeting distinct molecular targets and acting through different mechanisms disrupt the endoplasmic reticulum–mitochondria interaction in cardiomyocytes, leading to calcium overload, mitochondrial dysfunction, and mitochondrial damage, resulting in cardiomyocyte injury. Proteomics studies revealed that SIRT7 is a common cardiotoxicity target for these TKIs. Upregulating SIRT7 to counteract MCU activation significantly mitigates TKI-induced damage to endoplasmic reticulum–mitochondrial interactions and protects against cardiomyocyte injury (Figure 10).

**FIGURE 10:**
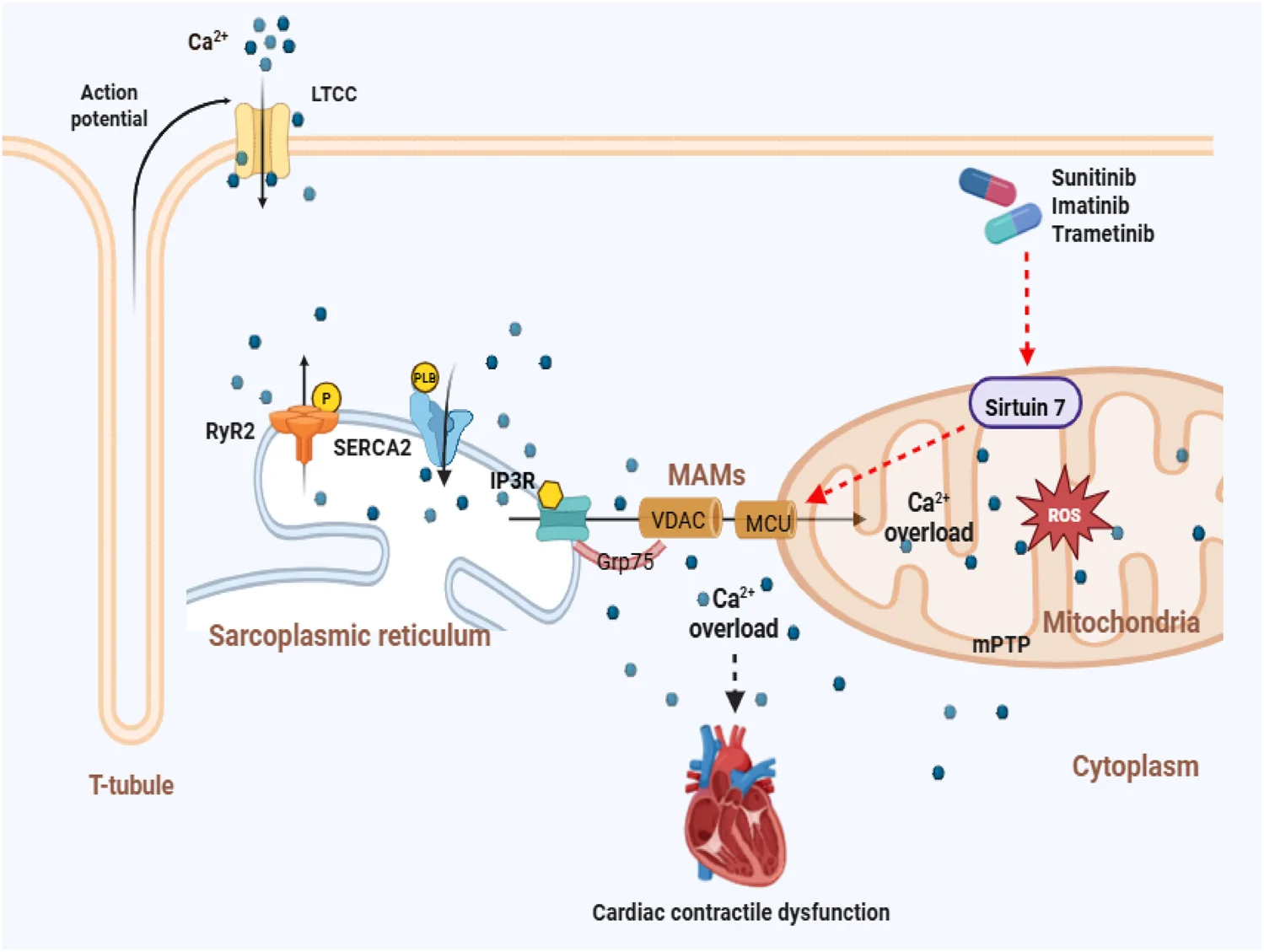
SIRT7 alleviates TKI-Induced cardiotoxicity via inhibition of MCU-Dependent mitochondrial Ca^2+^ overload.

## Data Availability

The original contributions presented in the study are included in the article/Supplementary Material, further inquiries can be directed to the corresponding author.
